# An assistant workforce to improve screening rates and quality of care for older patients in the emergency department: findings of a pre- post, mixed methods study

**DOI:** 10.1186/s12877-018-0811-6

**Published:** 2018-05-30

**Authors:** Carolyn Hullick, Jane Conway, Isabel Higgins, Jacqueline Hewitt, Bernadette Stewart, Sophie Dilworth, John Attia

**Affiliations:** 1 The University of Newcastle, University Drive, Callaghan, NSW 2308 Australia; 2 Hunter Medical Research Institute, Locked Bag 1000, New Lambton, NSW 2305 Australia; 3 School of Health, University of New England and Conjoint Professor of Nursing, University of Newcastle, Newcastle, Australia; 4 School of Nursing and Midwifery, University of Newcastle, Newcastle, Australia; 5 Aged Care Emergency Service, Clinical Nurse Consultant, Patient Flow Unit, Hunter New England Local Health District, Newcastle, Australia; 6 John Hunter Hospital, Hunter New England Health, Locked Bag 1, HRMC, Armidale, NSW 2310 Australia

**Keywords:** Emergency services, hospital, Comprehensive geriatric assessment, Emergency nursing, Caregivers, Delirium, Standard of care, Assessment, nursing, Older person, Screening, Delirium prevention, Emergency department, aged

## Abstract

**Background:**

Older people who present to the Emergency Department (ED) experience high rates of prevalent and incident delirium. This study aimed to determine whether an assistant workforce in the ED could effectively conduct screening to inform assessment and care planning for older people as well as enhance supportive care activities for prevention of delirium.

**Methods:**

Using a pre-post design, data was collected before and after the introduction of Older Person Technical Assistants (OPTAs) in the ED. OPTA activity was recorded during the intervention period and a medical record audit undertaken prior to and 9 months after implementation.

Data were analysed using descriptive statistics for OPTA activities. Weighted Kappa scores were calculated comparing concordance in screening scores between OPTAs and Aged Services Emergency Team Registered Nurses. Changes in the rates of documented screening and supportive care were analysed using Chi-square tests.

Focus groups were conducted to explore clinicians’ experiences of the OPTA role.

**Results:**

Three thousand five hundred fourty two people were seen by OPTAs in 4563 ED Presentations between 1st July 2011 and 2012. The reproducibility of all screening tools were found to be high between the OPTAs and the RNs, with Kappas and ICCs generally all above 0.9.

The medical record audit showed significant improvement in the rates of documented screening, including cognition from 1.5 to 38% (*p* < 0.001) and review of pain from 29 to 75% (*p* < 0.001). Supportive care such as being given fluids or food also improved from 13 to 49% (*p* < 0.001) and pressure care from 4.8 to 30% (*p* < 0.001). This was accomplished with no increase in ED length of stay among this age group.

Focus group interviews described mixed responses and support for the OPTA role.

**Conclusions:**

An assistant workforce in an ED setting was found to provide comparable screening results and improve the rates of documented screening and supportive care provided to older people with or at risk of developing delirium in the ED. There is a need for a shared philosophy to the care of older people in the ED.

**Trial registration:**

Australian New Zealand Clinical Trials Registration number is ACTRN12617000742370. It was retrospectively registered on 22nd May 2017.

## Background

When older people present to the Emergency Department (ED) they often require immediate support to ensure their safety as well as other needs in the ED environment [1–4]. Providing early review of individual patient risk, comprehensive assessment and a tailored management plan minimises the risks associated with ED presentation and admission to hospital as well as the risk of ED re-presentation [5, 6]. This paper reports on the introduction of an aged care assistant workforce in a tertiary referral hospital ED in New South Wales (NSW), Australia. Titled the Older Person Technical Assistant (OPTA), the assistant position aimed to optimise the quality of care for older people in ED and reduce preventable adverse events, particularly delirium [7]. The use of a non-professional workforce to screen and provide supportive care for delirium management and prevention as well as facilitate access to comprehensive geriatric assessment for older people who present to the ED has not previously been examined.

Delirium is an acute change in mental state. It is characterised by disturbance of consciousness, attention, cognition and perception [8, 9]. With older age, particularly those over 75 years of age, there is a higher risk of developing delirium in hospital. About 10% of older people already have prevalent delirium on admission to hospital [10], with a further 8% of older people developing delirium during their hospitalisation [10, 11]. Delirium is associated with poorer hospital outcomes including increased length of stay, falls risk, pressure injuries and functional decline. This can lead to older people requiring admission to nursing homes, as well as premature death [12–15]. Despite these known concerns, delirium is often under-recognised and under-diagnosed [9, 16–18]. Prevention of delirium along with effective management when it occurs, improves health outcomes [19, 20] as well as cost effectiveness [21].

Predisposing and precipitating factors for developing delirium have been identified [22]. These include pre-existing dementia, sensory impairment, being acutely unwell and institutional care as well as infection, decreased mobility or the use of restraints, bladder catheterisation, malnutrition, dehydration, more than three medications, and sleep deprivation [14, 15, 22].

In Australia, the Australian Commision for Quality and Safety introduced a Delirium Clinical Care Standard in 2016 including early screening for delirium, interventions to prevent delirium as well as falls, and pressure injury prevention [8]. High quality care of older people in hospital requires delirium prevention and management [21–25].

## Methods

### Aim

The study aimed to determine whether an assistant workforce in the ED could effectively conduct screening to inform assessment and care planning of older people as well as provide supportive care activities for prevention of delirium.

### Design

This study used a pre-post design, where data were gathered before and after the introduction of Older Person Technical Assistants (OPTAs) in the ED.

### Setting

The setting was a tertiary referral hospital Emergency Department in NSW, Australia. In the year prior to this study, the ED had 67,000 patient presentations, 13% were patients over 75 years of age.

### Intervention

The intervention was four full time equivalent OPTAs working 8 h shifts between 8 am to 8 pm in the ED, 7 days a week for 12 months. OPTAs focused on screening and the supportive care of people over the age of 75 years who were not experiencing life threatening situations. The prerequisites for selection as an OPTA included previous training or experience as a health care assistant or equivalent. On commencement in the ED, over a 5-day period, they were oriented to the environment, trained and assessed as competent in tasks performed by assistants in nursing, maintaining patient privacy and dignity, and using relevant screening tools. They were instructed about how to record screening findings and supportive care in the electronic patient management system.

**Fig. 1:**
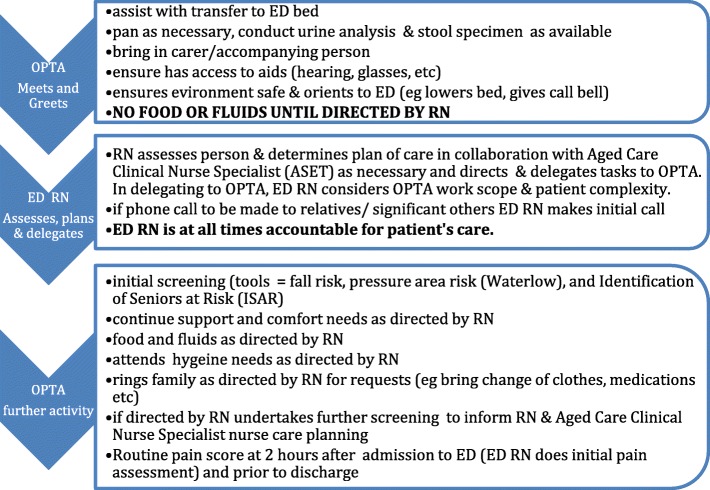
Flow of tasks for the Older Person Technical Assistant role in the ED

The role of the OPTA in screening and supportive care under the delegation and supervision of a Registered Nurse (RN) was documented in a care protocol (Fig. 1). The OPTA was able to independently assist with the transfer of the patient onto the ED bed and bring the patient’s carer into the ED, as well as orient the patient and their families to the ED environment. Any further care or assessment required delegation from the ED RN.

Screening undertaken included:

- Six item screener, a brief cognitive assessment screen designed for EDs [23, 24];
- Confusion Assessment Method Instrument (CAMI) for delirium [25, 26];
- Falls Risk for Hospitalised Older People, a falls screening tool [27];
- Identification of Seniors at Risk, a tool to identify patients at risk for ED representation [28];
- Numerical rating scale for pain [29];
- Waterlow screening tool for pressure injury risk screening [30];
- Mini-Nutritional Assessment for nutrition [31]; and
- Modified Care Strain Index to screen for caregiver strain in long-term family caregivers [32].

In line with the Australian clinical practice guidelines for the management of people with delirium, preventative environmental and clinical practice strategies were incorporated into the role [7, 8, 19, 33]. This multicomponent delirium prevention strategy consisted of orienting the person to the ED environment, attending to nutrition, hydration and elimination needs, providing sensory and mobility aids, supervising and assisting moving, one-on-one support when agitated and facilitating carer presence with the patient as often as possible. A delirium prevention box that included daily newspapers, playing cards, games and puzzles, crosswords, a radio with ear phones and a clock was available for OPTAs to use with patients [11]. OPTAs also provided support to carers in the ED who are often older people themselves.

### Data collection

Data collection included documentation of screening and supportive care provided by the OPTAs. Data was collected from 1st July 2011–2012, 3 months following the implementation of OPTAs in order to ensure the screening and care process was embedded as routine OPTA practice and there was accuracy and compliance in documenting activity. Data was recorded in the hospital administrative system by the OPTAs. This included screening and results as well as care activities that they provided.

### Focus groups

Given that screening and supportive care are an important part of delirium prevention and that the proposed model of care assumed the RN acted on screening results conducted by the OPTAs, six focus groups were undertaken with RNs in the ED to explore their experience of the introduction of the OPTA role.

### Data analysis

Screening scores obtained by OPTAs were compared with those obtained by Aged Services Emergency Team (ASET) RNs. These advanced clinical RNs target care for older patients with chronic and complex care needs in the ED [34].

The scores obtained by OPTAs and those obtained by RNs for each screening tool were compared using weighted Kappa; 95% Confidence Intervals were calculated using the Jackknife variance estimation method. In order to calculate correlation, a smaller group of patients was screened independently by one of the five OPTAs as well as one of the six RNs. To account for possible correlations between patients with the same OPTA/RN screening, a variable with the OPTA/RN identifier was added as a clustering variable.

Patients presenting the year before the OPTA Intervention, 1st July 2010 to 30th June 2011 were compared to patients seen by OPTAs 1st July 2011 to 30th June 2012. In order to determine the extent to which screening, assessment and care interventions known to prevent delirium and other adverse events in older people were documented as having been undertaken in the ED, the medical records of 63 patients were randomly selected for the medical record documentation audit, undertaken by members of the research team comparing the same period, 1st March 2010 to 30th June 2010 compared to 1st March 2012 and 30th June 2012. The medical record audit reviewed evidence of documentation for assessment of delirium risk factors and supportive care, including ongoing pain review, provision of food or fluids, orientation, toileting, mobilization, pressure care and family or carers in attendance with the patient. Results were compared using Chi-squared tests.

Focus group interviews were recorded and transcribed verbatim. Focus group data were analysed thematically. Significant statements were highlighted and clustered around the goals of the model of care.

### Ethical considerations

The Hunter New England Health Human Research Ethics Committee approved the study in February 2011, reference no. 11/02/16.5.01; HREC/10/HNE/402; SSA/10/HNE/402. Consent from individual patients was not required as data were routinely collected through administrative hospital data. No individuals were approached and all data were de-identified.

## Results

OPTAs saw 3542 people who presented to the ED on a total of 4563 occasions, as some patients had more than one ED visit over the 12 months. The mean number of visits per patient seen by OPTAs was 1.3 (SD = 0.7) and the maximum was 13. With a roster that covered 12 h each day, OPTAs saw 53% of patients aged over 75. This is further described in Table 1. ED length of stay was comparable between 2010/11 and 2011/12, *p* = 0.21, for all patients presenting over the age of 75 years. The OPTAs were less likely to see patients who were Australian Triage Category 1 as they were critically ill, requiring immediate resuscitation and Triage Category 5 as they have less urgent conditions and are usually ambulant and able to care for themselves in the ED. The patients who they saw were more likely to be admitted to hospital, reflecting a longer ED length of stay.

**Table 1:** Characteristics of patients over the age of 75 years seen in the ED from 1st July 2010 to 30th June 2011 compared to 1st July 2011 to 30th June 2012 and those seen by the OPTA within the ED between 1st July 2011 and 1st July 2012

Characteristic	2010/11	2011/12	Subset Seen by OPTA (2011/12)
Total number of patient presentations over the age of 75 years	8455	8287	4563 (53%)
Number of individual patients	5835	5667	3542
Age in years: Mean (SD)	83 (5)	84 (5)	84 (5)
Age in years: Median (min, max)	83 (75,101)	83 (76,101)	83 (76, 101)
ED Length of stay in minutes: Mean (SD)	455 (265)	450 (257)	524 (278)
ED Length of stay in minutes: Median (min, max)	415 (3,2691)	412 (5,1899)	465 (41, 1931)
Australasian Triage Category: 1-Resuscitation	97 (1.1%)	95 (1.1%)	23 (< 0.1%)
2-Emergency	1156 (14%)	1068 (13%)	579 (13%)
3-Urgent	2719 (32%)	2761 (33%)	1753 (38%)
4-Semi-Urgent	3999 (47%)	3940 (48%)	2113 (46%)
5-Non-urgent	484 (6%)	423 (5%)	95 (2.1%)
Discharged from ED	5776 (68%)	5660 (68%)	1161 (25%)
Admitted to hospital	2679 (32%)	2627 (32%)	3402 (75%)

Medical records of 63 patients were reviewed prior to the introduction of the OPTAs. The findings are presented in Table 2. It reveals a lack of documentation of screening results and care delivery, compared with the same period after the introduction of the OPTA. There was a significant improvement in documentation of all areas of care and screening.

**Table 2:** Screening Tools and delirium Prevention Strategies documented prior and after the OPTA introduction

Problem screened or patient care intervention	Pre-OPTA introduction (1st March 2010 to 30th June 2010) *N* = 63	Post OPTA (1st March 2012 to 30th June 2012) N = 63	*p*-value (Chi-sq test)
Problem Screened			
Pain	51 (81%)	53 (84%)	0.6
Falls	29 (46%)	42 (67%)	0.02
Pressure Injury risk	26 (41%)	43 (68%)	0.002
Nutrition	1 (1.5%)	26 (41%)	< 0.001
Cognition	1 (1.5%)	24 (38%)	< 0.001
Pain review	18 (29%)	47 (75%)	< 0.001
Medication review	2 (3.2%)	29 (46%)	< 0.001
Comprehensive assessment	31 (49%)	36 (57%)	0.3
Care intervention			
Given food or fluids	8 (13%)	31 (49%)	< 0.001
Orientation	0 (0%)	32 (51%)	< 0.001
Toileting	0 (0%)	21 (33%)	< 0.001
Mobilisation	0 (0%)	26 (41%)	< 0.001
Pressure care	3 (4.8%)	19 (30%)	< 0.001
Family or carers in attendance	1 (1.5%)	18 (29%)	< 0.001

The results of the screening by OPTAs are presented in Table 3. The reproducibility of all screening tools were found to be very high between the OPTAs and the RNs, with Kappas and ICCs generally all above 0.9.

**Table 3:** Screening undertaken by the OPTAs and inter-rater agreement with RN

Tool and Statistic	Results
Six Item Screener	
Number of patients screened by OPTAs (N)	2347 (51%)
Number of patients screened by RN for validation of six Item screener (N)	86
Weighted Kappa (95% CI)	0.95 (0.90, 0.99)
Confusion Assessment Method Instrument (CAMI)	
Number of patients screened by OPTAs (N)	1199 (26%)
Number of patients screened by RNs for validation of CAMI (N)	58
Weighted Kappa (95% CI)	0.88 (0.60, 1.00)
Identification of Seniors at Risk	
Number of patients screened by OPTAs (N)	2593 (57%)
Number of patients screened by RN for Validation of Identification of Seniors at Risk (N)	73
Weighted Kappa (95% CI)	1.00(0.99, 1.00)
Falls Risk Screen	
Number of patients screened by OPTAs (N)	3969 (80%)
Number of patients screened by RNs for validation of falls risk screen by RN (N)	99
Weighted Kappa (95% CI)	0.95 (0.89, 1.00)
Pain Score (0 to 10)	
Number of patients screened by OPTAs (N)	2921 (64%)
Number of patients screened by RN for validation of pain score by RN (N)	62
Weighted Kappa (95% CI)	0.91 (0.70, 1.00)
Carer Strain Index	
Number of patients screened by OPTAs (N)	547 (12%)
Number of patients screened by RN for validation of carer strain index (N)	20
Weighted Kappa (95% CI)	1.00 (1.00, 1.00)
Mini-nutritional Assessment	
Number of patients screened by OPTAs (N)	1697 (37%)
Number of patients screened by RN for validation of mini-nutritional assessment (N)	61
Weighted Kappa (95% CI)	1.00(1.00, 1.00)
Waterlow Pressure Ulcer Risk Screen	
Number of patients screened by OPTAs (N)	3665 (80%)
Number of patients screened by RNs for validation of Waterlow Pressure Ulcer Risk Screen (N)	81
Weighted Kappa (95% CI)	0.90(0.78, 1.00)

Table 4 outlines the delirium prevention supportive care tasks undertaken and recorded by the OPTAs, under the direction of the patient’s ED RN responsible for the patient’s care. After assessment, the RN determined whether patients were safe to eat and drink or walk to the bathroom, depending on their usual level of function and the acute illness that precipitated their ED presentation. Patients requiring one-on-one supportive care were patients who were calling out, distressed or at risk of falling out of bed. If patients were unable to walk to the bathroom, OPTAs assisted with toileting at the bedside. Orientation and general care were the most frequent activities at 67 and 39% respectively.

**Table 4:** Delirium prevention activities undertaken by OPTAs

Delirium prevention activities	Total (*N* = 4563)
Specials, one-on-one supportive care	104 (2%)
General care	1782 (39%)
Mobility assistance	1183 (26%)
Assistance with meals and fluids	1410 (31%)
Assistance with toileting	1172 (26%)
Orientation	3036 (67%)
Sensory assistance such as access to glasses and hearing aids	1531 (34%)
Therapeutic activities such as reading the paper.	397 (9%)

### Focus groups with registered nurses

Fifty ED staff participated in six focus groups. Fourty-seven were RNs including three ASET RNs. The remaining three were a social worker, pharmacist and dietician. Eight RNs were from short stay inpatient wards where some older people went following their ED visit. There was consensus in the focus groups that OPTAs made a positive contribution to care of older people though there was limited discussion of OPTA screening translating into communication, decision-making and care planning.

### RNs, OPTAs and ASET staff working together to screen older people

Some RNs indicated that the screening results undertaken by the OPTAs were not considered as part of their care planning. They felt the screening results were not for ED RNs but for other nurses whose role it was to provide specific assessment for older people:

*As RNs we do our own assessment… The tools are probably useful at a later stage of the hospitalisation or for community follow up but in the ED we are focused on different things (Registered Nurse)*

*Pain is among the main symptoms people have when they come to ED. It is the RNs’ role to manage that and that requires the RN assesses pain. (Registered Nurse)*

Other RNs, in contrast to this, explained that the screening results provided useful information for guiding ongoing assessment and care planning:

*It all probably affects ASET [*Aged Services Emergency] *team more than RN in ED and has lessened ASET load I would imagine. (Registered Nurse)*

*Well I have noticed that the falls risks are signposted. (Registered Nurse)*

*I have found that we have a better sense of the person and their needs when they arrive from ED. I use the screens to guide what we need to be doing and to inform discharge planning (ASET Registered Nurse)*

ASET nurses described the usefulness of the OPTA screening, assisting them in targeting specific older patients for assessment in the ED, as they do not have time to assess all older patients and could focus on those with higher needs. They reported that OPTAs used the scores along with their general impressions and concerns when they discussed the patients with the ASET Nurses. They also felt that screening facilitated engagement in issues by the medical staff. For example the CAMI results indicated the need to address delirium and its cause and management. The ASET nurses reported that OPTAs screening for delirium, identified a number of positive results, which escalated the care of these older people in the ED earlier than would have happened prior to the OPTA role.

### OPTAs providing supportive care

The supportive care undertaken by OPTAs was primarily intended to prevent delirium as well as positively impact the experience for older people and their caregivers in the ED, who are often older themselves. Despite this, some RNs viewed the OPTA activity as “nice” to have rather than essential, particularly for delirium management, as reflected in the following comments:

*It is nice the oldies have a cup of tea and something to eat now. Prior to the OPTAs we were all too busy to give those little extras that make such a difference. (Registered Nurse)*

*Having the family member made to feel welcome is important to them. (Registered Nurse)*

However ASET nurses, as well as some other RNs, viewed the care provided by OPTAs as a delirium prevention strategy.

## Discussion

The Australian Commission on Safety and Quality in Healthcare Delirium Clinical Care Standard [8] provides guidance for appropriate care and reducing unwarranted variation by defining the care that patients can be expected to be offered or receive. It outlines seven focus areas for acute hospitals for delirium, including early screening; early assessment; early interventions to prevent delirium; early identification for the underlying cause; prevention of other hospital risks, pressure injuries and falls given their higher risk for patients with delirium; minimisation of the use of anti-psychotics for patients with delirium; and better transition home for patients with delirium. The challenge for EDs is having prevention and management of delirium recognised as a priority, in competition with other pressing demands. The OPTA role supports RNs to deliver better quality patient care in many of the seven focus areas within the Delirium Clinical Care Standard. Informed by nursing assessment and screening results, OPTAs delivered care that had regularly not previously occurred in the ED, like access to meals and fluids. Having a dedicated non-professional workforce with a limited scope of practice that focused on older patients, allowed for earlier responsiveness to the needs of older people, supporting delirium prevention. In patients with delirium the OPTA role assisted with the prevention of falls and pressure injuries through supportive care as well as providing one-on-one care for patients who were agitated and distressed, aiming to reduce the need for anti-psychotic medication in an ED environment that is disturbing [11]. The purpose of OPTAs was primarily to focus on improving screening and care intervention for delirium management in the ED but there was secondary opportunity for broader improvements in comprehensive assessment and care. This was dependent on the extent to which others in the care team framed OPTA work as essential screening that informs other assessment and care processes rather than seeing OPTA work as adding ‘niceties’ to an ED environment.

The validation results demonstrate that the OPTAs were able to perform screening tests in the ED with the Kappas generally above 0.90 with the screen for delirium; CAMI at 0.88 is also a high correlation. OPTAs were also able to deliver high levels of supportive care that promotes delirium prevention. Having an assistant workforce that was focused on delirium prevention highlight to other staff that delirium prevention was a high clinical priority. The documented rate of screening and supportive care improved significantly across all areas. Staff in the ED reported greater awareness of falls risk in the ED with more systematised screening by the OPTAs.

All older patients did not require interventions and screening. If patients presented to the ED with minor problems and were discharged home from the ED they did not need OPTA support. If they presented to ED out of the hours of the OPTA, they were not seen. Patients who were screened and had care interventions by OPTAs were much more likely to be admitted to hospital; it is not clear whether this was a consequence of the identification of more issues by the OPTAs or that the OPTAs were instructed by RNs to focus on those with more higher needs. OPTAs documented significantly higher rates of family and carers in attendance in the ED though it still remained low at 29%. Having OPTAs undertaking screening meant that the ASET RNs did not need to undertake this activity and were able to use information from OPTAs to determine which patients needed more comprehensive assessment.

Previous research suggests that multicomponent strategies delivered by a trained, non-professional group of health care workers such as the OPTAs, are effective in reducing incidence, duration and number of episodes of delirium [7, 35, 36]. This study has demonstrated that an assistant workforce can accurately use screening tools and record findings, as well as assist with supportive care of patients and their families, without any increase in the overall ED length of stay for this group. We were not able to determine the impact of the OPTAs on the prevention and management of delirium. An assumption in this study was that delivering preventative care and screening more reliably with a dedicated workforce such as the OPTAs enhances quality care and prevents delirium although this could not be directly measured in our audit.

Care strategies that prevent delirium and other risks associated with ED presentation are foundational to quality patient experience and healthcare. Assessment, monitoring and provision of patient care needs including access to hydration and food, mobilisation, orientation and reorientation, ensuring contact with family and supporting family are important pillars of excellent nursing care [37–40]. Prioritising these care needs, guided by a shared philosophy and clarity in roles and responsibilities is essential for improving the ED experience for older people.

Limitations of the design were that delirium and other outcomes such as falls and pressure injuries were not measured. It is thought, however, that the activity undertaken by the OPTAs is likely to have reduced these known risks for older people in the ED.

It is more important to ensure that delirium prevention strategies are embedded in care responsibilities within the ED rather than a single workforce group. The OPTA raised the profile for the need for supportive care of older people in the ED with scope of practice limited and focused on their care. OPTAs assisted in raising the profile of delirium prevention and management within the ED that had not been previously prioritised. Documentation gaps for aspects of care of older people in the ED were identified in this study. If delirium prevention strategies are to be prioritised, documentation of these strategies as well as improved documentation of delirium and other undesired consequences of hospitalisation needs to occur.

The findings of this study highlight that a non-professional workforce, the OPTAs, can undertake screening in the ED and support the management and prevention of delirium, however, the uptake of the screening provided by OPTAs and including a dedicated workforce focused on older people in the model of care in the ED is dependent upon the extent to which other groups, particularly RNs accept and include OPTA screening in decision making without replicating the screening.

## Conclusions

Older people in the ED require screening in order to ensure risk factors are identified and addressed. Preventative strategies in ED require dedicated staff, screening embedded in care, comprehensive and systematic assessment, management and documentation of care and ongoing treatment. The Older Person Technical Assistant can support this.

## Abbreviations

ASET: Aged service emergency team
CAMI: Confusion Assessment Method Instrument
ED: Emergency Department
FTE: Full time equivalent
OPTA: Older Person Technical Assistant
RN: Registered Nurse
